# Optimizing antimicrobial therapy in urinary tract infections: A focus on urine culture and sensitivity testing

**DOI:** 10.3389/fphar.2022.1058669

**Published:** 2022-11-30

**Authors:** Rama Alkhawaldeh, Rana Abu Farha, Khawla Abu Hammour, Eman Alefishat

**Affiliations:** ^1^ Department of Clinical Pharmacy, Faculty of Pharmacy, Applied Science Private University, Amman, Jordan; ^2^ Department Biopharmaceutics and Clinical Pharmacy, Faculty of Pharmacy, The University of Jordan, Amman, Jordan; ^3^ Department of Pharmacology, College of Medicine and Health Science, Khalifa University of Science and Technology, Abu Dhabi, United Arab Emirates; ^4^ Center for Biotechnology, Khalifa University of Science and Technology, Abu Dhabi, United Arab Emirates

**Keywords:** urinary tract infection, urine culture, antimicrobial, Jordan, sensitivity tests

## Abstract

**Objectives:** This cross-sectional study was conducted at Jordan university hospital to evaluate the impact of microbial culture data and sensitivity results on optimizing UTI treatment.

**Methods:** All positive urine cultures requested for adult patients (≥18 years) admitted to Jordan University Hospital (JUH) within the period from January 2019–July 2021 were evaluated. The antibiotics prescribed before and after culture data and sensitivity results were compared to evaluate the impact of these diagnostic measures on optimizing UTI treatment.

**Results:** During the study period, 2400 urine cultures revealed positive results. Among those patients, 1,600 (66.7%) were discharged before the availability of culture results and excluded. Of the remaining 800 patients, 701 patients (87.6%) received empiric treatment. After culture and sensitivity results were available, overall, 84 (10.5%) patients had optimization (improvement) in their UTI management after culture results were known, while 6 (0.8%) patients had a worsening in their treatments. Based on the culture results, we found that only 12.4% of patients were appropriately treated before and after the culture results. Moreover, our results revealed that 31.9% were inappropriately treated for their UTIs before and after culture results.

**Conclusion:** This study revealed an alarmingly high rate of inappropriate treatment of UTIs despite the availability of urine culture and sensitivity data, and that culture results were not used to optimize treatment strategies for UTI. This practice can potentially result in poor health-related outcomes and adversely affects efforts to battle AMR. Multifaceted strategies must be implemented to help clinicians follow the best current evidence and current guidelines in their selection of antibiotics for the management of UTIs.

## Introduction

Optimum therapeutic antimicrobial management should target a specific pathogen with a precise dose and treatment duration in order to effectively combat causative microbes, reduce the risk of complications, minimize adverse drug reactions, and lower the risk of antimicrobial resistance (AMR) (Niederman, 2003). It has been reported that in order to efficiently optimize antimicrobial use and reduce the risk of antimicrobial resistance, healthcare providers should follow a multiple-step approach to reduce the risk of AMR (Graber et al., 2015). This approach includes different points of assessment for the appropriateness, dose, duration, and route of administration of antimicrobials. This assessment is especially important after microbial culture results become available, commonly after the initiation of empiric treatment, to evaluate the sensitivity of the identified pathogen (Kinn et al., 2018). Following these assessments has been concluded as a strategy to help better manage infections, reduce adverse drug reactions, and unnecessary exposure to antimicrobial agents, which can potentially help control AMR (Wolfe et al., 2019).

The optimal use of antimicrobial agents in hospitalized patients includes a correct selection of empiric antimicrobials agents as well as the targeted agents (Carson and Naber, 2004). Inappropriate prescribing not only results in negative health-related outcomes but also increases the risk of AMR. According to the Centers for Disease Control and Prevention (CDC), more than 2.6 million people get infected with antimicrobial-resistant microorganisms each year in the United States, resulting in about 44,000 deaths as a minimum (Dadgostar, 2019).

Irrational and excessive use of antibiotics has been documented by different studies in Jordan (Shehadeh et al., 2012; Yusef et al., 2018; Alsayed et al., 2022). Self-medication of antibiotics without a doctor’s prescription is one example of irrational usage, which can contribute to the development of bacterial resistance toward the antimicrobial agents (Al-Azzam et al., 2007). According to previous studies, the prevalence of irrational use of antibiotics in Jordan was approximately 40.7%, which is considered significant (Al-Azzam et al., 2007; Sawair et al., 2009; Yusef et al., 2018). The effectiveness of antimicrobial agents decreases with time depending on the frequency of use, this feature makes them different than other drug classes (Llor and Bjerrum, 2014; Reygaert, 2018). Thus, all healthcare professionals must use the currently available antimicrobials rationally and prescribe them properly in order to avoid going back to the era before the discovery of antimicrobials (Alanis, 2005).

Several investigations have been conducted to assess the pattern of antimicrobial resistance in Jordan (Abdullah and Shara, 2011; Shakhatreh et al., 2019). A study conducted by Abdullah et al. showed that 90.9% of Klebsiella pneumonia isolated from different clinical specimens were resistant to imipenem (Abdullah and Shara, 2011). Furthermore, another study found that 81.9% of *E. coli* isolates from urine cultures exhibited resistance to at least three different types of antimicrobials (Shakhatreh et al., 2019). However, the need to implement Antimicrobial stewardship (AMS) programs in Jordan has arisen because millions of people are infected annually with antimicrobial-resistant organisms and tens of thousands of them die. AMS programs are among the most effective strategies to overcome bacterial resistance *via* taking various actions aimed to directly influence antibiotic use and reduce unnecessary antibacterial prescriptions (Dyar et al., 2017). Therefore, AMS is an important approach that must be applied in all hospitals, regardless of their size (Stenehjem et al., 2017).

Urinary tract infections (UTIs) are common infectious diseases at the community level that attack any region of the urinary system (Amawi et al., 2021). The prevalence of UTIs in females is 30 times higher than in males under the age of 50 (Amawi et al., 2021). Complicated UTIs (those infections in immunocompromised patients, males, and those associated with anatomical abnormalities) are often hard to treat and caused mainly by a diverse species of gram-negative and gram-positive bacteria, increasing antimicrobial resistance, and a higher prevalence of recurrent infections. The most common causative microbes are gram-negative bacteria, including Escherichia *coli* (*E. coli*), followed by *Klebsiella* and *Proteus* species (Foxman, 2010). Patients with complicated UTIs most likely require empiric broad-spectrum intravenous antimicrobial therapy (O’Grady et al., 2019). Early detection and confirmation of the causative organisms by culture and sensitivity testing is of critical importance for the management of UTIs. Urine culture remains the gold standard for UTIs investigation, and antimicrobial therapy should be tailored based on the results of the urine culture (Tan and MPJSmj, 2016). This is of special significance in order to improve therapeutic outcomes in treated patients, minimize side effects, and help combat AMR. Thus, the aim of this study is to evaluate the impact of microbial culture data and sensitivity results on optimizing UTI management in a tertiary teaching hospital in Jordan.

## Materials and methods

### Study design, participants, and data collection

This is a retrospective cross-sectional study that was conducted at Jordan University Hospital (JUH), Amman-Jordan, all urine cultures requested for patients (≥18 years) admitted to JUH between January 2019–July 2021 were reviewed, and only patients with positive culture were considered.

Following patients’ identification, information regarding urine culture and sensitivity testing were obtained from JUH laboratory electronic system. Data on the prescribed empiric antimicrobials were collected from patients’ medical records among other clinical and demographic data. Any change in the selection of antimicrobials following the urine culture and sensitivity results were also documented.

### Study outcomes

Urinary tract infection was considered to be appropriately treated empirically with antimicrobial if the identified microorganism, as per the microbial culture results, was within the spectrum covered by that empiric antimicrobial, and if the organism was reported as susceptible to that antimicrobial agent (Harvey et al., 2012). UTIs were flagged as inappropriately treated if any of the following was documented; having no antimicrobial therapy prescribed “untreated”, being treated with antimicrobial that does not cover the identified microorganism “lack of coverage”, and being treated with antimicrobial that was reported as “resistant”. In some cases, it was not possible to judge the appropriateness of treatment due to the lack of sensitivity testing. In a similar way, the appropriateness of treatment following culture results was evaluated.

After that, antimicrobials prescribed before and after culture and sensitivity testing were compared to evaluate the appropriateness of the management of UTIs. The treatment of UTIs was either 1) improved (treatment was inappropriate before culture and became appropriate after culture results), 2) worsened (treatment was appropriate before culture and became inappropriate after culture results), 3) not changed since the patients were treated appropriately before and after culture and sensitivity testing, or 4) not changed since the patients were treated inappropriately before and after culture and sensitivity testing.

### Ethical consideration

The World Medical Association Declaration of Helsinki guidance was followed in the study (World Medical, 2013). The study was initiated after obtaining approval from the Institutional Review Board (IRB) committee at JUH which is the teaching hospital affiliated with the University of Jordan (Reference No. 196/2021). Patient informed consent was waived by the ethics committee due to the retrospective nature of the study. All the collected information was kept on the personal computer of the principal investigator using password-protected files.

### Statistical analysis

All the collected data were coded, entered, and analyzed using the Statistical Package for Social Sciences (SPSS) version 22. The descriptive analysis was conducted usingmedian/interquartile range (IQR) f continuous variables, while frequency and percentages were used for categorical variables. Checking for normality was carried out using the Shapiro-Wilk test (with *p* ≤ 0.05 indicating that our continuous variables were not normally distributed). McNemar’s test was carried out to evaluate differences in antimicrobials appropriateness rate before and after obtaining culture results. A *p* ≤ 0.05 was considered statistically significant. All tests were two-tailed.

## Results

### Demographic and medical characteristics of the study sample

During the study period, urine cultures were ordered for 6,950 patients, 4,550 (65.5%) patients tested negative and were excluded from the study and only those with positive culture were included in the study (*n* = 2,400, 34.5%). Among those patients with positive culture episodes, 1,600 patients (66.7%) were discharged too early before the availability of culture results and were also excluded which left us with a total sample of 800 patients.

The median age of participants (*n* = 800) was 64 years (IQR = 29), with 71.0% of the participants (*n* = 568) being above 50 years old, and more than two-thirds of them were females (*n* = 555, 69.4%). Moreover, more than half of the patients (*n* = 437, 54.6%) were receiving polypharmacy (defined as ≥4 medications), and they had a median length of hospital stay of 12 days (IQR = 11). For more details about the demographic and medical characteristics of the study sample, refer to Table 1.

**TABLE 1:** Demographic and medical characteristics of the study sample (*n* = 800).

Parameter	Results
Age in years, median (IQR)	64.0 (29.0)
Age categories (years), *n* (%)	
20–50	232 (29.0)
50.1–80.0	483 (60.4)
80.1–110	85 (10.6)
Gender, *n* (%)	
Female	555 (69.4)
Male	245 (30.6)
Number of chronic medications, *n* (%)	
0–1	178 (22.3)
2–3	185 (23.1)
≥4	437 (54.6)
Length of stay, median (IQR)	12.0 (11.0)

IQR: interquartile range.

### Antimicrobial prescription before and after culture results

Antimicrobials were primarily prescribed empirically before culture results for 701 patients (87.6%), while 12% of the patients (*n* = 99) did not receive any empiric antimicrobial. The median number of the prescribed empiric antimicrobials for all of the recruited patients was 1.0 (IQR = 0.0), with a total of 873 prescribed empiric antimicrobials. The most frequently prescribed empiric antimicrobials were ceftriaxone (*n* = 214, 24.5%), imipenem/cilastatin (*n* = 209, 23.9%), and levofloxacin (*n* = 128, 14.7%).

**FIGURE 1:**
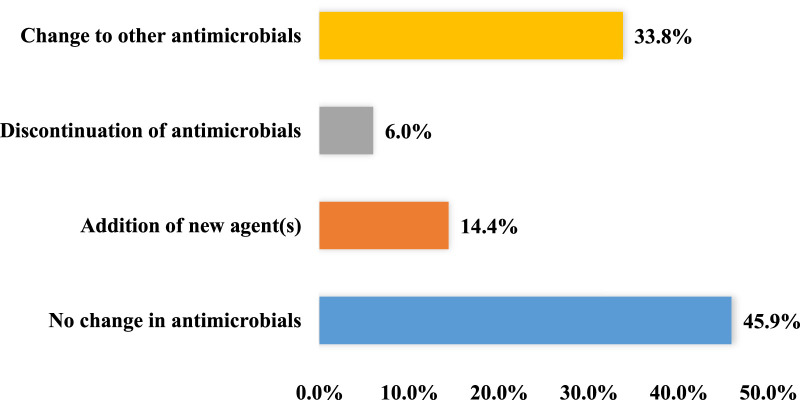
Changes in antimicrobial following culture and sensitivity testing (*n* = 800).

Following culture and sensitivity testing, 367 (45.9%) patients had their antimicrobials continued, 270 (33.8%) had them changed, 114 (14.4%) had additional agents to have targeted antimicrobial coverage, 48 (6.0%) patients had agents discontinued (Figure 1). Following the availability of culture results, patients received a total of 972 antimicrobials. These agents were prescribed for 743 patients (92.9%), while 57 patients (7.1%) received no antimicrobial and were flagged as “untreated”. The most frequently prescribed antimicrobial following culture results were imipenem/cilastatin (*n* = 276, 28.4%), levofloxacin (*n* = 115, 11.8%), and ceftriaxone (*n* = 105, 10.8%).

### Urine culture and sensitivity testing

Most urine culture specimens revealed one microorganism (*n* = 559, 69.9%), with few specimens showed two or more pathogens (241, 30.1%). The most frequently reported pathogens were *E. coli* (*n* = 313, 29.5%), *Enterococcus* (*n* = 189, 17.8%), and *Staphylococcus* (*n* = 158, 14.9%). Less than half of the patients had their sensitivity testing reports performed (*n* = 391, 48.9%). There were 136 instances of resistance to antimicrobials out of the 873 prescribed empiric antimicrobials (15.6%). These antimicrobials reported as “resistant” were prescribed for 117 patients out of the 800 eligible patients (14.6%). Ceftriaxone (*n* = 48, 35.3%), levofloxacin (*n* = 27, 19.9%), and imipenem/cilastatin (*n* = 21, 15.4%) were the main empiric antimicrobials that with reported resistence.

### The impact of culture and sensitivity testing on optimizing antimicrobials prescribing

**FIGURE 2:**
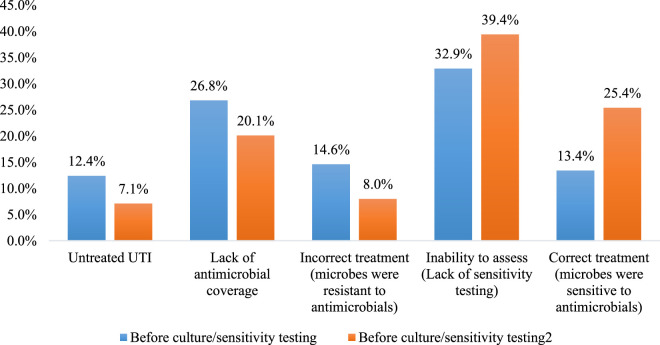
The difference between the appropriateness of antimicrobials before and after culture and sensitivity testing (*n* = 800). *p* < 0.001 using McNemar test.

The difference between the appropriateness of antimicrobials before and after culture and sensitivity testing is presented in Figure 2. The number of untreated patients was reduced from 99 (12.4%) to 57 (7.1%) following culture and sensitivity testing. Moreover, the “lack of coverage” was reduced from 214 (26.8%) to 161 (20.1%). Also, the incorrect treatment (the identified pathogens were reported as resistant to the prescribed antimicrobial) was reduced from 117 (14.6%) to 64 (8.0%). Finally, the number of patients with correct treatment was increased from 107 (13.4%) to 203 (25.4%), *p* < 0.001.

**FIGURE 3:**
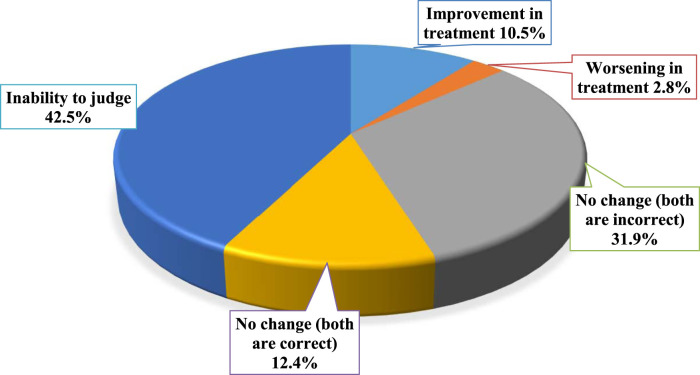
The impact of urine culture and sensitivity testing (if available) on optimizing antimicrobials prescribing (*n* = 800).

The impact of urine culture and sensitivity testing on optimizing antimicrobials prescribing was evaluated and the results are presented in Figure 3. Results showed that the treatment of 10.5% of the patients (*n* = 84) was improved, while only 0.8% of the patients (*n* = 6) had less appropriate treatment (worsening). The remaining patients have no change in the appropriateness of their treatments, where 99 patients (12.4%) have received correct antimicrobials before and after culture and sensitivity testing, while 255 patients (31.9%) have received inappropriate antimicrobials before and after culture and sensitivity testing. The impact of antimicrobial changes could not be assessed for 356 patients (44.5%) since sensitivity testing was not performed to patients. Several examples of the impact of urine culture and sensitivity testing on optimizing antimicrobials prescribing were presented in Table 2.

**TABLE 2:** Examples of the effect of antimicrobial adjustment following culture and sensitivity results.

The effect of adjustment	Example
Improvement in antimicrobials prescribing	A 41 Years old female admitted to JUH, results of urine culture showed the presence of MRSA. The patient was given Micafungin as an empiric antimicrobial before results of culture are available. This antimicrobial was not correct because of the lack of coverage. The specific antimicrobial prescribed following obtaining culture results was changed to Vancomycin. The effect of adjustment according to culture result and sensitivity test is considered appropriate
	A 53 Years old female admitted to JUH, results of urine culture showed the presence of *E. coli*. The patient was given Levofloxacin as an empiric antimicrobial before results of culture are available. This antimicrobial was not correct because of the resistance. The specific antimicrobial prescribed following obtaining culture results was changed to Imipenem/cilastatin. The effect of adjustment according to culture result and sensitivity test is considered appropriate
Worsening in antimicrobials prescribing	A 77 Years old male admitted to JUH, results of urine culture showed the presence of *E. coli*. The patient was given Imipenem as an empiric antimicrobial before results of culture are available. According to culture result and sensitivity test this antimicrobial is considered correct. The specific antimicrobial prescribed following obtaining culture results was changed to Levofloxacin. The effect of adjustment according to the sensitivity test is considered inappropriate
	A 59 Years old female admitted to JUH, results of urine culture showed the presence of *E. coli*. The patient was given Levofloxacin as an empiric antimicrobial before results of culture are available. According to culture results and sensitivity test this antimicrobial is considered correct. No antimicrobial was prescribed following obtaining culture results and sensitivity test- (Untreated). The effect of adjustment is considered inappropriate
No change in antimicrobials prescribing (both correct)	An 80 Years old female admitted to JUH, results of urine culture showed the presence of *E. coli*. The patient was given Imipenem as an empiric antimicrobial before results of culture are available. The prescribed antimicrobial was correct according to culture result and sensitivity test. The specific antimicrobial prescribed following obtaining culture results was changed to Meropenem. Both antimicrobials are considered correct
	A 79 Years old female admitted to JUH, results of urine culture showed the presence of *E. coli*. The patient was given Piperacillin/Tazobactam as an empiric antimicrobial before results of culture are available. The prescribed antimicrobial was correct according to culture result and sensitivity test. The specific antimicrobial prescribed following obtaining culture results was not changed. The antimicrobial is considered correct
No change in antimicrobials prescribing (both incorrect)	An 80 Years old female admitted to JUH, results of urine culture showed the presence of *Klebsiella*. The patient was given Ceftriaxone as an empiric antimicrobial before results of culture are available. The prescribed antimicrobial was not correct because of the resistance. The specific antimicrobial prescribed following obtaining culture results was changed to Cefuroxime. The effect of adjustment according to results of sensitivity test is considered inappropriate
	A 36 Years old female admitted to JUH, results of urine culture showed the presence of *Pseudomonas*. The patient was given Ceftriaxone as an empiric antimicrobial before results of culture are available. The prescribed antimicrobial was not correct because of the lack of coverage. The specific antimicrobial prescribed following obtaining culture results was changed to Imipenem/Cilastatin. The effect of adjustment according to results of sensitivity test is considered inappropriate

## Discussion

This study assessed the impact of urine culture and sensitivity testing in optimizing UTI treatment for 800 hospitalized patients in Jordan. According to our observations, UTIs management was improved in 10.5% of patients when results of culture and sensitivity testing were available. Several studies in the literature have supported the importance of microbial culturing and sensitivity tests in guiding the appropriate antimicrobial therapy (Reller et al., 2009; Bayot and Bragg, 2019; Benkova et al., 2020). Sensitivity tests are of special importance for the appropriate management of infection, they help guide physicians in determining which antimicrobials are most likely to be effective in combating microbial growth (Khan et al., 2019).

The improvement in UTI management reported in this study included treating those who were untreated prior to the availability of culture results. The number of patients with untreated UTIs decreased from 12.4% to 7.1% with the availability of culture results. These results indicate a delay in antimicrobial therapy which can potentially increase morbidity and the risk of complications of UTIs. Despite the need to control antimicrobial resistance and refrain from prescribing antimicrobial agents unless necessary, delay in antimicrobial therapy has been reported to negatively impact patients health-related outcomes and adds to the economic burden of the management of UTIs (Lodise et al., 2019). Rapid diagnostic testing has been recommended to guarantee appropriate and timely use of antimicrobial agents, this can help improve patient outcome and reduce the risk of antimicrobial resistance (Reuter et al., 2019).

Unfortunately, about one-third of patients (31.9%) still inappropriately treated even after culture results are available. Moreover, six (0.8%) patients had a worsening in their treatments after the results of the culture were available. Our results are consisted with the conclusion of a recent review where a change to targeted antimicrobial therapy occurred in 50% of patients in 22 hospitals in the Netherlands, however, only 32% of the changes were correct (Hulscher et al., 2017). Antibiotic stewardship to support appropriate antibiotic prescribing patterns is essential to provide efficient and cost effective treatment minimizing the risk of complications and antimicrobial resistance (Hulscher et al., 2017).

Furthermore, we were unable to assess the impact of urine culture and sensitivity testing on optimizing UTI treatments in 44.5% of the patients due to the lack of sensitivity reports which indicate that microbiological sensitivity testing is not being carried out as recommended for all patients. Previous studies stated that the resistance pattern of uropathogens in UTI patients changes over time, which mandates special attention and monitoring to decrease the risk of therapeutic failure and microbial resistance (Linhares et al., 2000; Akram et al., 2007). Recent studies have shown the value of culture and sensitivity testing in decreasing inappropriate antibacterial use (Tabak et al., 2018; Maugeri et al., 2019). The value of performing microbiological sensitivity testing over culture alone was assessed in Denmark by Holm et al. (2015). Because of the rising prevalence of resistance, they predicted that treating enterococci based on culture would result in 20%–30% inappropriate antibiotic therapy. In contrast, sensitivity testing should increase proper antibiotic prescriptions by more than 90%. Another study conducted in Nicaragua (Latin America) concluded that the choice of antimicrobial therapy should be based on the results of sensitivity testing to decrease the possibility of resistance and the emergence of ESBL producing species (Bours et al., 2010). These results support what was previously proposed regarding the importance of sensitivity testing in tracking antibiotic resistance levels, increasing appropriate treatment, and improving patient outcomes.

In this study, we identified a significant number of patients who were excluded from the study (66.7%) with pending lab tests, where results of culture were obtained after discharge. Unfortunately, lab results showed positive urine culture in those patients. A similar finding was observed in a study conducted by Roy et al. (2005), which has identified a considerable proportion of patients (41%) that were discharged too early before the availability of culture results, and 12.6% of them required immediate action to initiate or modify the antibiotic therapy, which could result in negative consequences in patient outcomes. They observed that this was especially relevant at tertiary care academic hospitals since primary care physicians and inpatient physicians were frequently unaware that a test had been requested (Roy et al., 2005). Another study conducted by Walz et al. (2005) reported a high number (32%) of pending lab tests at a single academic medical facility in the Unites States. These findings emphasize the importance of developing reliable interventions to improve the communication of pending lab tests at discharge between hospital laboratory facilities and inpatient and outpatient providers, and to ensure follow-up (Roy et al., 2005; Walz et al., 2011).

This study has several limitations. In this study, the lack of data on patient’s medical history can influence the physician’s choice of antibiotic, dosage, and duration of therapy. In addition, patients’ data were obtained from a single Jordanian tertiary center’s (JUH) database, which limits the generalizability of the conclusion. Multicenter studies covering different regions of Jordan should be conducted to confirm these findings. Also, the evaluation of the appropriateness of the UTI empiric treatment was judged based on the empirical treatment and the diagnosis only, without knowing the history of the patient or what drove the physician to prescribe a particular drug. Moreover, without knowing a patient medical history, it was not possible to evaluate the quality of the prescription according to the clinical practice guidelines. However, this is the first study to evaluate the impact of urine culture and sensitivity testing on optimizing UTI treatment in Jordan and taking into consideration the observational design of the study, it is considered satisfactory to provide background data at this stage.

## Conclusion

This study revealed an alarmingly high rate of inappropriate treatment of UTI even after the availability of urine culture and sensitivity data, and that culture results were not used to optimize treatment strategies for UTI. This may increase the risk of therapeutic failure and microbial resistance. We also report a high rate of lack of sensitivity testing which is equally important and can potentially lead to increased risk of complications and antimicrobial resistance. Multifaceted strategies including antibiotic stewardship programs must be implemented to enhance clinicians’ appropriate management of UTIs starting from ordering the right diagnostic tests ending with the appropriate selection of antibiotics for treatment of UTI which should be guided by culture and sensitivity tests results.

## Data Availability

The original contributions presented in the study are included in the article/supplementary material, further inquiries can be directed to the corresponding author.
